# Adaptative Mechanisms of Halophytic *Eutrema salsugineum* Encountering Saline Environment

**DOI:** 10.3389/fpls.2022.909527

**Published:** 2022-06-28

**Authors:** Chuanshun Li, Chonghao Duan, Hengyang Zhang, Yaoyao Zhao, Zhe Meng, Yanxiu Zhao, Quan Zhang

**Affiliations:** ^1^Shandong Provincial Key Laboratory of Plant Stress Research, College of Life Science, Shandong Normal University, Jinan, China; ^2^Research Team of Plant Pathogen Microbiology and Immunology, College of Life Science, Shandong Normal University, Jinan, China

**Keywords:** *Arabidopsis* relative model system, salt cress, salt stress tolerance, saline adaptation, antioxidant system, osmo-adaptation, ion homeostasis, gene expression

## Abstract

Salt cress (*Eutrema salsugineum*), an *Arabidopsis*-related halophyte, can naturally adapt to various harsh climates and soil conditions; thus, it is considered a desirable model plant for deciphering mechanisms of salt and other abiotic stresses. Accumulating evidence has revealed that compared with *Arabidopsis*, salt cress possesses stomata that close more tightly and more succulent leaves during extreme salt stress, a noticeably higher level of proline, inositols, sugars, and organic acids, as well as stress-associated transcripts in unstressed plants, and they are induced rapidly under stress. In this review, we systematically summarize the research on the morphology, physiology, genome, gene expression and regulation, and protein and metabolite profile of salt cress under salt stress. We emphasize the latest advances in research on the genome adaptive evolution encountering saline environments, and epigenetic regulation, and discuss the mechanisms underlying salt tolerance in salt cress. Finally, we discuss the existing questions and opportunities for future research in halophytic *Eutrema*. Together, the review fosters a better understanding of the mechanism of plant salt tolerance and provides a reference for the research and utilization of *Eutrema* as a model extremophile in the future. Furthermore, the prospects for salt cress applied to explore the mechanism of salt tolerance provide a theoretical basis to develop new strategies for agricultural biotechnology.

## Introduction

Soil salinity is one of the major environmental stress factors that restrict the functioning of plants in natural ecosystems, and greatly reduce crop yields which subsequently leads to a threat to food security (Zhu, 2002; Vera-Estrella et al., 2005; Zhao et al., 2020). As we know, the enormous value will come from a better understanding of the mechanisms through which plant tolerance of abiotic stresses is achieved (Zhao et al., 2020). Most studies on salt stress response mechanisms of plants have been conducted using the glycophyte model *Arabidopsis thaliana* and some important clues were gained (Wu et al., 2012). However, *Arabidopsis* has a relatively low capacity to survive salt stress, thus it may lack the protective mechanisms required for growing in harsh environments (Lee et al., 2013). Therefore, searching for the novel stress tolerance determinants including novel genes and novel stress tolerance mechanisms has led to increasing interest in the plants native to growing in extreme environments called “extremophytes” (John and Spangenberg, 2005; Amtmann, 2009; Gechev et al., 2012; Bressan et al., 2013; Oh et al., 2013; Cheeseman, 2015; Flowers et al., 2015; Eshel et al., 2017).

**Figure 1:**
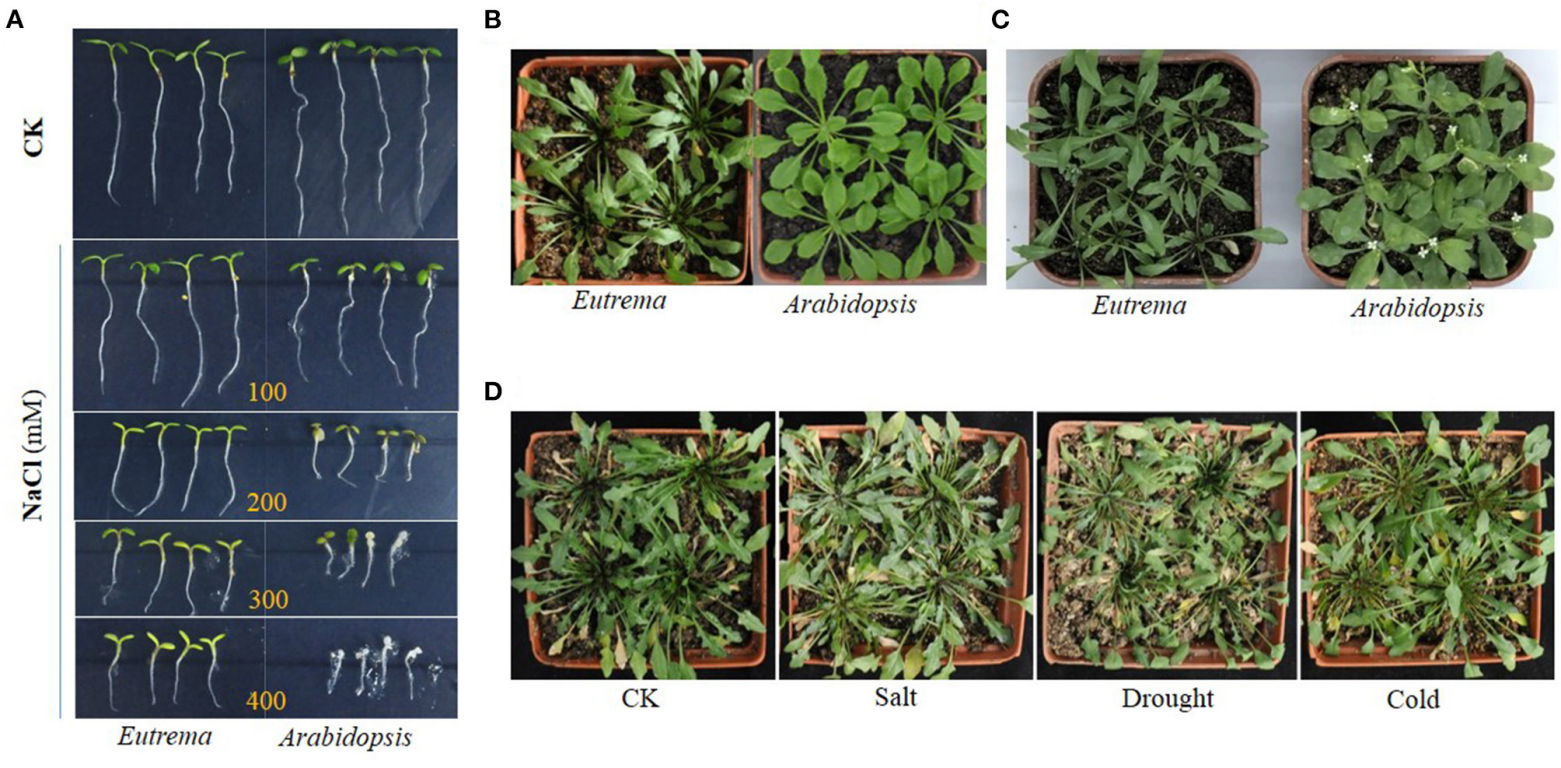
Growth comparison of *Eutrema salsugineum* (Shandong) and *Arabidopsis thaliana* plants in the control (CK) and stress treatment conditions. **(A)** Growth comparison of *Eutrema* and *Arabidopsis* plants in the control and NaCl treatment (100, 200, 300, and 400 mM NaCl) conditions. *Eutrema* seeds germinated 10 days, and *Arabidopsis* seeds germinated for 7 days in 1/2 MS medium, then the seedlings of uniform growth moved to 1/2 MS medium containing 0 (CK), 100, 200, 300, and 400 mM NaCl for growing for about 5 days. **(B)** Growth comparison of the 50-day-old *Eutrema* and 30-day-old *Arabidopsis* plants that grow in a mixture of vermiculite, perlite and peat moss (1:1:1), with a photoperiod of 16 h light/22°C and 8 h dark/18°C. **(C)** Growth comparison of the 60-day-old *Eutrema* and 40-day-old *Arabidopsis* plants. The surface sterilized *Eutrema* seeds planted in 1/2 MS medium, was firstly stratified treatment (4°C for 13 days) to synchronize the germination, then moved into the growth chamber for 7 days; the seedlings were transplanted in nutrient soil for 34 days, followed by 6 day-salt treatment using 200 mM NaCl. The surface sterilized *Arabidopsis* seeds planted in 1/2 MS medium, was firstly stratified treatment in 4°C for 3 days, then moved into a growth chamber for 7 days; the seedlings were transplanted in nutrient soil containing vermiculite, perlite, and peat moss (1:1:1) for 24 days, followed by 6-day salt treatment using 200 mM NaCl. **(D)** Phenotype comparison of *Eutrema* plants under salt, drought, and cold stresses. *Eutrema* seeds were planted in a mixture of vermiculite, perlite, and peat moss (1:1:1) in a greenhouse for 60 days with a photoperiod of 16 h light/22°C and 8 h dark/18°C, followed by stress treatments using 300 mM NaCl (Salt), no watering (Drought) and low temperature of 4–6°C (Cold) for 2 weeks, respectively, or kept in normal growth condition (CK).

Halophytes can naturally thrive under high salinity conditions, such as in marshlands, swamps, and intertidal estuarine areas, and their tolerance to salt stress may occur through various evolutionary and molecular mechanisms, which can provide crucial insights into the underlying mechanisms that endow tolerance of plants to salt stress (Flowers et al., 1986; Bressan et al., 2001; Zhu, 2002; Amtmann, 2009; Rajalakshmi and Parida, 2012; Wu et al., 2012). The crucifer *Eutrema salsugineum* (salt cress) formerly classified as *Thellungiella salsuginea* or *Thellungiella halophila* is a close relative of *Arabidopsis*. *Eutrema salsugineum* can naturally grow well in extreme environments and displays exceptionally high resistance to salt as well as cold, drought, and oxidative stresses, but its genetic make-up, morphology, and development are similar to the glycophyte *Arabidopsis* (Bressan et al., 2001; Inan et al., 2004; Taji et al., 2004; Volkov et al., 2004; Wang et al., 2004; Gong et al., 2005; Wong et al., 2006; Amtmann, 2009; Orsini et al., 2010; Wu et al., 2012; Koch and German, 2013; Figure 1). Furthermore, salt cress is an ideal model organism due to its short life cycle, self-fertility, and being genetically transformable, and has a relatively small genome (241 Mb) with approximately twice the genome size of *Arabidopsis* (Inan et al., 2004; Wu et al., 2012). Importantly, salt cress has the availability of extensive ecotypes exhibiting a range of stress responses to adapt to different environmental stresses (Inan et al., 2004; Wong et al., 2005; Li et al., 2006; Amtmann, 2009; Hou and Bartels, 2015). These advantages characteristics make the halophytic *Eutrema* species amenable to the functional genomics approaches designed to identify the novel genes and new molecular mechanisms involved in stress resistance (Lugan et al., 2010).

Over the past few years, this salt cress species has come to the fore in the research into abiotic stress resistance and is considered a plant model of stress resistance well comparable with the *Arabidopsis* genome (Bressan et al., 2001; Inan et al., 2004; Amtmann et al., 2005; Amtmann, 2009). Firstly, the genome of salt cress provides a resource for identifying naturally occurring genetic alterations which could contribute to the adaptation of the halophyte to soil salinity, and that might be bioengineered in related crop species (Yang et al., 2013). The characterization of transcripts with unknown functions will possibly reveal the novel defense mechanisms in this halophyte (Rajalakshmi and Parida, 2012). Secondly, comparative analyses of the genome structures, protein-coding genes, microRNAs, stress-related pathways, and proteomic and metabolic profiles between *Eutrema* and *Arabidopsis* suggest that adaptation of the halophyte to environmental salt stress may occur *via* a global network adjustment of multiple regulatory mechanisms (Yang et al., 2013). Furthermore, the research of *Eutrema* is supported by growing technical resources including the physiological and molecular protocols, ecotype collections, and comparison of genome, transcriptome, and metabolome. Hence, recent scientific advances make exploration of the mechanisms of environmental adaptation in plants more feasible which could result in a larger implication and gains in genetic improvement of crops.

In this review, we systematically summarize the recent research advances in understanding morphology, physiology and biochemistry, expression and expression regulation of genes, and evaluating its usefulness as a model for research into plant stress tolerance, as well as emphasizing the possible molecular mechanisms underlying adaptation of the halophytic *Eutrema* to saline environments. This can provide a comprehensive insight to understand the mechanisms conferring a high level of salt stress tolerance in the halophyte.

## Genome Evolution Adapted to Saline Environment

High levels of stress tolerance in plants are the result of evolutionary adaptation, thus the genotypic selection of plants for adaptation to various environments plays an important role in agriculture and ecology (Boyer, 1982; Janz et al., 2010). Due to the natural adaptations of *E. salsugineum* to various harsh climates and soil conditions, it is considered as an important model for deciphering the mechanisms of salt and other abiotic stress tolerance of plants (Gong et al., 2005; Griffith et al., 2007; Lamdan et al., 2012; Wang et al., 2021).

Sixteen accessions of halophytic *E. salsugineum* species including previously called *T. salsuginea, T. halophila*, and *T. botschantzevii* were used to investigate their natural variation in salinity tolerance and found that these accessions could survive up to 700 mM NaCl in hydroponic culture, however, their relative salt tolerance has a considerable variation (Lee et al., 2016). Using 90 resequencing whole genomes data from 90 individuals in 21 natural populations, the analysis of *Eutrema* species across central Asia to North America revealed that the selection signals for genes were related to the adaptation of salt and other abiotic tolerance, at the species level (Wang et al., 2021).

Gene duplication, a major source of genetic diversity that drives the adaptive evolution of plants (Ohno, 1970; Kondrashov, 2012; Monihan et al., 2019). Wu et al. (2012) demonstrated that, although the gene spaces display an extensive colinearity between *E. salsugineum* and *A. thaliana*, the salt stress-related gene families expanded in *E. salsugineum* genome (Wu et al., 2012). For instance, 21 transcription factor (TF) families expanded in the *E. salsugineum* genome, compared to *A. thaliana*. Among these, the *AtNFXL1* gene encoding NF-X1 type zinc finger proteins was suggested to play a regulatory role in protecting photosynthesis, and be required for the growth of *Arabidopsis* plants under salt stress. The members of *NF-X1, HSF, Trihelix*, and *GRAS* TF families involved in abiotic stress response, were also expanded in numbers in salt cress (Table 1). The expansions of these gene family members are suggested to be associated with the adaptation of salt cress to harsh environments, due to the fact that their orthologous gene members in *Arabidopsis* and other plants have been documented to be associated with stress resistance (Lisso et al., 2006; Ogawa et al., 2007; Xie et al., 2009; Wu et al., 2012). These expanded family members possibly endow *E. salsugineum with* more flexibility in response to salinity stress (Wu et al., 2012). Additionally, the *RAV* gene family reported to respond to high salt and cold stresses, had been expanded from six members in *A. thaliana* to nine in *E. salsugineum* genome (Fowler et al., 2005; Sohn et al., 2006; Wu et al., 2012; Table 1). However, the *AtRAV1* gene overexpressed plants showed strong growth retardation. And comparing to the wild type, a more inhibition of seed germination in salt conditions, and the transgenic plants exhibited higher transpirational water loss in drought conditions (Fu et al., 2014). Given the expressions of *RAVs* in *Arabidopsis* were reduced by salt and dryness, it is inferred that adaption of reduced *AtRAV1* expression is involved in the adaption of salinity environment in *Arabidopsis* (Fu et al., 2014). Moreover, the transcripts of *EsRAVs* in the group A (A-EsRAVs) from *E. salsugineum* seedling exhibited a moderate decline gradually by salt treatment, and stronger inhibition of seed germination and seedling root elongation occurred in the *35S:A-EsRAV* transgenic plants in presence of NaCl, suggesting the roles of *35S:A-EsRAVs* in negatively controlling plant growth (Yang et al., 2016; Table 1).

**Table 1:** The expanded genes or gene family members involved in response to salt stress in *Eutrema* compared with *Arabidopsis*.

**Gene family**	**Number of genes or gene family members**		***Eutrema***		***Arabidopsis***		**Function of genes**	**References**
	***Eutrema***	***Arabidopsis***	**Gene name**	**Gene ID**	**Gene name**	**Gene ID**		
CBL10	2	1	EsCBL10a	Thhalv10026019m	AtCBL10	AT4G33000	Calcium-mediated signaling capacity; Salt tolerance	Monihan et al., 2019
			EsCBL10b	Thhalv10028908m				
HKT1	3	1	EsHKT1;1	Thhalv10028767m	AtHKT1	AT4G10310	Na^+^/K^+^ co-transporter	Ali et al., 2012
			EsHKT1;2	Thhalv10028594m				Ali et al., 2013
			EsHKT1;3	Thhalv10028595m				
LEA	42	40	EsEM1	Thhalv10010923m	AtEM1	AT3G51810	Salt and drought tolerance	Xiang and Man, 2018
			EsLEA1	Thhalv10014897m	AtLEA4-5	AT5G06760	Salt tolerance	Zhang et al., 2012
NFXL	3	2	EsNFXL1	Thhalv10012642m	AtNFXL1	AT1G10170	Salt tolerance	Lisso et al., 2006
HSF	28	24	EsHSF2	Thhalv10002333m	AtHSF2	AT2G26150	Salt and osmotic stress tolerance; callus growth	Ogawa et al., 2007
RAV	9	6	EsRAV1	Thhalv0010019m	AtRAV1/EDF4	AT1G13260	*Arabidopsis* RAVs are negative regulators of growth and salt stress; *Eutrema* A-EsRAV inhibits seed germination and seedling root elongation; Pepper CaRAV1 is involved in bacterial infection resistance and osmotic tolerance	Sohn et al., 2006
			EsRAV2	Thhalv0019566m	AtRAV2/EDF2	AT1G68840		Fu et al., 2014
			EsRAV3	Thhalv0004508m	AtRAV3/EDF3	AT3G25730		Yang et al., 2016
			EsRAV4	Thhalv0007983m	AtRAV4/EDF1	AT1G25560		
			EsRAV5	Thhalv0012152m	AtRAV5	AT1G51120		
			EsRAV6	Thhalv0012161m	AtRAV6	AT1G50680		
			EsRAV7	Thhalv0012356m				
			EsRAV8	Thhalv0012377m				
			EsRAV9	No found				
Trihelix	31	29	EsGT2	Thhalv10018336m	AtGT2	AT1G76890	Salt, freeezing and drought tolerance	Xie et al., 2009; Fang et al., 2010
GRAS	40	33	EsCBF1	Thhalv10027405m	AtCBF1	AT4G25490	Freezing tolerance; growth-repressing	Achard et al., 2008

LEA proteins can be involved in the “molecular shield function” to protect enzymes from induced aggregation when plants encounter stress in their environments (Goyal et al., 2005). In *E. salsugineum, LEA* genes were found to be higher copy numbers than their orthologous genes in the *Arabidopsis* genome, which is in agreement with a higher frequency of gene duplications observed in the *E. salsugineum* and *E. parvula* genomes (Table 1). This indicated that, since the last whole genome duplication event or after tandem duplications leading to neofunctionalization, the two *Eutrema* species (*E. salsugineum* and *E. parvula*) retained a higher fraction of duplicated genes, which is regarded as an important adaptive strategy for salt cress to survive in habitats with more extreme environmental conditions than *Arabidopsis* (Blanc and Wolfe, 2004a,b; Maere et al., 2005; Dassanayake et al., 2011; Wu et al., 2012; Lee et al., 2013; Oh et al., 2013).

The salt overly sensitive (SOS) pathway functions in preventing the toxic accumulation of sodium in the cytosol when *A. thaliana* are grown in salt-affected soils. In this pathway, *Arabidopsis* AtCBL10 calcium sensor interacts with the AtSOS2 kinase to activate the AtSOS1 plasma membrane sodium/proton exchanger, which initiates the transport of sodium out of the cell (Shi et al., 2000; Qiu et al., 2002, 2003; Quintero et al., 2002). In *E. salsugineum*, the *CBL10* gene had been duplicated into *EsCBL10a* and *EsCBL10b* (Table 1), and the down-regulation of either of the two *EsCBL10* genes decreased the growth of plants in the presence of salt, indicating that both genes function in the response of plants to salinity stress (Monihan et al., 2019). Further, *EsCBL10b* was demonstrated to have an enhanced ability to activate the SOS pathway, while the role of EsCBL10a is in an alternative pathway with different functions from *AtCBL10* or *EsCBL10b*. Taken together, the duplication of *Eutrema EsCBL10* obviously increased calcium-mediated signaling capacity and conferred an enhanced salt tolerance when compared with salt-sensitive Arabidopsis (Monihan et al., 2019).

Altogether, the maintenance of duplicated gene pairs in the genome of plants is indicative of an adaptive benefit conferred by the paralogous genes (Hahn, 2009; Kondrashov, 2012; Monihan et al., 2019). Undoubtedly, genetic variation and natural selection are fostering stress-related genes and gene interaction networks in the whole genome, which will prompt the local adaptation and differentiation of *Eutrema* plants to extreme salt stress in a non-random way.

## Morphological Characteristics and Physiological Responses

The exposure of plants to high salt stress leads to the damage of cell structure, and disorder of many physiological functions (Sairam and Tyagi, 2004). As a consequence, this will eventually result in photosynthesis inhibition and metabolic impairment to further impaired growth and fertility, and early senescence of plants (Larcher, 2003).

**Figure 2:**
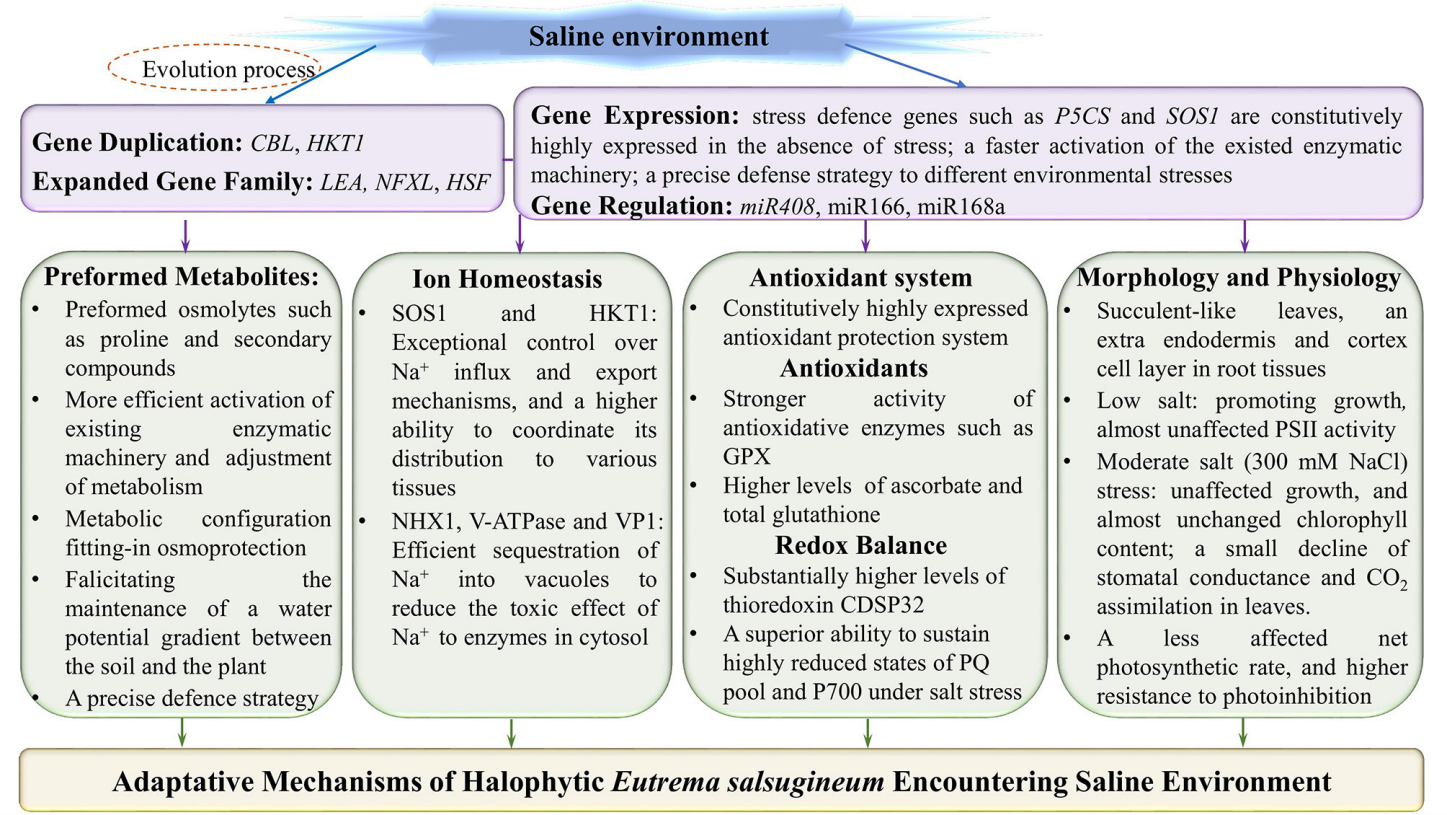
Adaptative mechanisms of halophytic *Eutrema salsugineum* encountering saline environment. The adaptative mechanisms of *Eutrema* plants to saline stress are mainly including: (1) The adaptive evolution endowing *E*. *salsugineum* plants with the expanded defense related genes and/or gene family members to facilitate plants more flexibility in response to salinity stress; (2) The constitutive higher expression of stress defense genes such as *P5CS* and *SOS1* in the absence of salt stress; (3) Anticipatory prepared osmolytes such as proline and secondary compounds, and a higher efficience to activate the existed enzymatic machinery as well as the adjustment of metabolism; (4) The halophytic *E*. *salsugineum* possessing exceptional control over Na^+^ influx and export mechanisms, the superior ability to coordinate its distribution to various tissues, and the efficient sequestration of Na^+^ into vacuoles; (5) A constitutively upregulated antioxidative protection system, and the effective regulation of redox status in halophytic *Eutrema*; (6) The succulent-like leaves, an extra endodermis and cortex cell layer in root tissues, and higher density of stomata exhibitting a more tightly closing when responding to salt stress, as well as an almost unaffected net photosynthetic rate, in the lower or even moderate concentration of salinity. SOS1, salt overly sensitive 1 (plasma membrane Na^+^/H^+^ antiporter); NHX, vacuolar Na^+^/H^+^ antiporter; V-ATPase, vacuolar H^+^-ATPase; VP1 (V-Ppase), vacuolar H^+^-translocating inorganic pyrophosphatase (vacuolar H^+^-pyrophosphatase); HKT1, group 1 high-affinity K^+^ transporter; GPX, glutathione peroxidases; PSII, photosystem II; PQ, plastoquinone.

Salt cress can natively grow in extreme salinity environments, and reproduce after exposure to 500 mM NaCl (Inan et al., 2004). Under treatments of 100 and 200 mM NaCl, an increased growth was observed in *E. salsugineum* accompanied by almost unchanged fresh and dry weights (Figures 1, 2), whereas a significant growth reduction in *Arabidopsis*. In a moderate salinity of 300 mM NaCl, *E. salsugineum* plants grew rapidly accompanied by almost unaffected chlorophyll content, a small decline of stomatal conductance and CO_2_ assimilation in leaves, indicating the photosynthetic apparatus was little influenced by this salt concentration (Sui and Han, 2014; Figure 2). However, the growth of Arabidopsis was remarkably decreased by the 100 and 200 mM NaCl treatments, which was associated with strong suppression in both leaf initiation and leaf expansion (M'rah et al., 2007; Sui and Han, 2014). Compared to *Arabidopsis, E. salsugineum* leaves are more succulent-like, have a second layer of palisade mesophyll cells, and frequently fall off during extreme salt stress, while root tissues develop an extra endodermis and cortex cell layer (Inan et al., 2004; Figure 2). Meanwhile, the stomata of *E. salsugineum* leaf surface are present at a higher density and less open than that in *Arabidopsis*, and exhibit a more tightly closing when responding to salt stress (Inan et al., 2004; Figure 2). Therefore, when compared with *Arabidopsis, E. salsugineum* can maintain a high water uptake and ion transport (Volkov et al., 2004; M'rah et al., 2006, 2007; Amtmann, 2009).

Photosynthesis is a primary process that is influenced by salinity stress, that mainly attributed to a decreased CO_2_ availability caused by diffusion limitations *via* the stomata and the mesophyll, or a generated secondary oxidative stress, which will seriously affect leaf photosynthetic machinery (Lawlor and Cornic, 2002; Flexas et al., 2004, 2007; Munns et al., 2006; Chaves et al., 2009). In leaves of *Arabidopsis*, the photosynthetic activity was suppressed after 15 days of salt treatment with higher than 50 mM NaCl, which could be explained by the stomata closure and dramatic shrinkage of the RubisCO pool. By contrast, *E. salsugineum* leaves still remained at 50% of the stomatal conductance of the control, and a negligible influence of RubisCO quantity, when exposed to the same salt-treated conditions (M'rah et al., 2007).

Chlorophyll (Chl) and PSII play a key role in the response of leaf photosynthesis to environmental stresses (Baker, 1991). When plants are subjected to 100 or 200 mM NaCl treatments, there are no remarkable changes in the chlorophyll content and Chl a/b ratio, as well as almost do not affect the PSII activity in *E. salsugineum* leaf (Figure 2), whereas a progressive decline exists in *Arabidopsis* leaf, which suggests this halophyte has effective mechanisms to protect photosystem against salinity (Sui and Han, 2014). In addition, *E. salsugineum* was found to have an increased chlorophyll a/b ratio accompanied by a higher effective photochemical quantum yield (Y_II_) value and lower non-photochemical quenching (NPQ) value, and a more active photosystem 1 (PS1) (Wiciarz et al., 2015).

All in all, these studies clearly demonstrate that, compared to *A. thaliana*, the *E. salsugineum* displays a relatively stable stomatal conductance and CO_2_ assimilation, higher chlorophyll content, Chl a/b ratio, and higher resistance to photoinhibition, as well as almost unaffected net photosynthetic rate and Fv/Fm (maximal photochemical efficiency of PSII; Sui and Han, 2014; Wiciarz et al., 2015; Figure 2), thus protecting photosystem activities to maintain normal growth of salt cress, in the lower or even moderate concentration of salinity.

## Maintain ION Homeostasis Under Saline Environment

To survive salt stress, plants must maintain a balance between sodium (Na^+^) and potassium (K^+^) ions, thus the homeostasis of Na^+^ and K^+^ is considered the most important mechanism to reducing NaCl stress in higher plants (Zhao et al., 2020). Halophytes can activate the protective mechanisms to prevent high Na^+^ accumulation in the cytosol and to maintain photosynthesis (Niewiadomska and Wiciarz, 2015; Bose et al., 2017; Kazachkova et al., 2018; Grigore, 2019).

**Figure 3:**
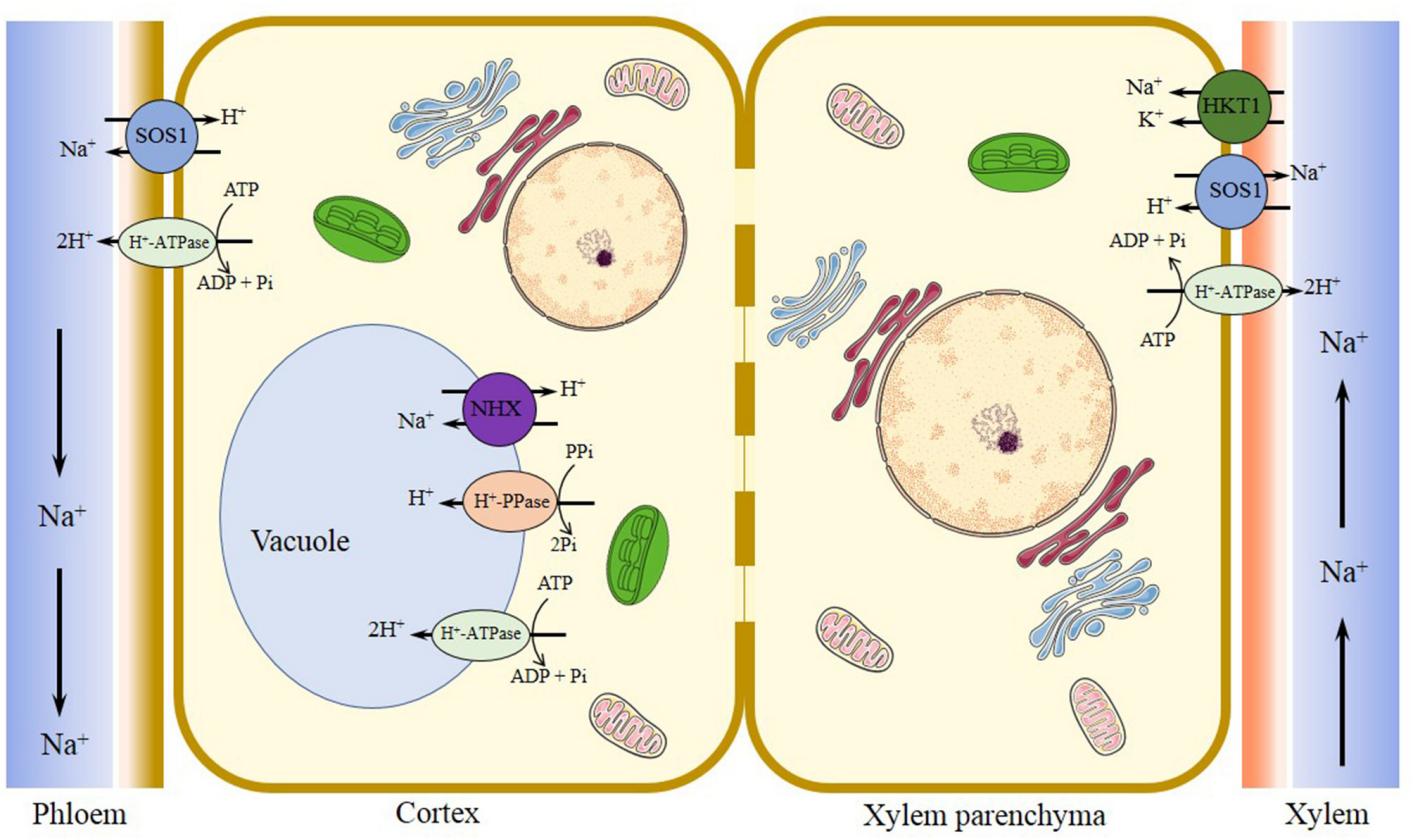
Major transporters regulating Na^+^*/*K^+^ homeostasis in *Eutrema* under salinized tissues. Plasma membrane Na^+^/H^+^ antiporter SOS1 functions in the regulation of ion homeostasis by extruding excessive Na^+^ out of cells, facilitating Na^+^ loading into the xylem in roots and shoots, and reducing the transfer of Na^+^ from roots to shoots, to counterbalance the uptake of Na^+^. Tonoplast-localized Na^+^/H^+^ exchanger NHX1 driven by vacuole H^+^-PPase (VP1) and/or H^+^-ATPase (V-ATPase) proton pumps is responsible for the sequestration of Na^+^ into the vacuole, to reduce the concentration of Na^+^ in the cytosol. The halophytic *Eutrema* HKT1;2 (EsHKT1;2 and EpHKT1;2) is considered as the Na^+^/K^+^ co-transporter, that function in Na^+^ retrieval from the xylem vessels under salt stress, and helps sequester ions into xylem parenchyma cells to protect photosynthetic tissues. SOS1, salt overly sensitive 1 (a plasma membrane Na^+^/H^+^ antiporter); NHX, vacuolar Na^+^/H^+^ antiporter; V-ATPase, vacuolar H^+^-ATPase; V-PPase, vacuolar H^+^-translocating inorganic pyrophosphatase (vacuolar H^+^-pyrophosphatase); HKT1, group 1 high-affinity K^+^ transporter.

The plasma membrane (PM) Na^+^/H^+^ antiporter (SOS1, Salt Oerly Sensitive 1) is involved in the transport of sodium ions across the plasma membrane and retrieves Na^+^ ions from the xylem sap, which is essential for Na^+^ and K^+^ homeostasis (Qiu et al., 2002; Shi et al., 2002). Taji et al. (2004) found, in *E. salsugineum*, a constitutively high expressed *EsSOS1* RNA in the absence of stress, and an increased *EsSOS1* expression observed in the PM of salt-treated roots and leaves (Taji et al., 2004; Vera-Estrella et al., 2005; Figures 2, 3). Likewise, Kant et al. (2006) demonstrated that *E. salsugineum* SOS1 transcript was strongly induced by salt in the shoots, and a constitutively high expression existed in roots in unstressed salt cress, which was consistent with a lower salt-induced Na^+^ concentration in xylem sap of *E. salsugineum* than that in *Arabidopsis* (Kant et al., 2006). Further, ectopic expression of *EsSOS1* from *E. salsugineum* suppressed the salt-sensitive phenotype of *Saccharomyces cerevisiae* strains lacking Na^+^ efflux transporters (SOS1), and enhanced the salt tolerance of wild-type *Arabidopsis* (Oh et al., 2009). *E. salsugineum* SOS1-RNA interference (RNAi) significantly induced reduction of *SOS1* gene, which led to faster leaf senescence and severe shoot water loss, accompanied by fragmentation of vacuoles, inhibition of endocytosis, and apoplastic Na^+^ influx into the stele and shoot (Oh et al., 2009). Another study also demonstrated that *EsSOS1* conferred greater salt tolerance than *AtSOS1* when expressed in a salt-sensitive strain of *S. cerevisiae*, and suggested that the salt tolerance of *E. salsugineum* may at least in part be derived from SOS1-mediated Na^+^ extrusion (Jarvis et al., 2014; Figures 2, 3). Therefore, it is suggested that EsSOS1 activity limits Na^+^ accumulation and affects the distribution of Na^+^, and acts as an important tolerance determinant in shaping the halophytic character of the *E. salsugineum* species (Oh et al., 2009; Figures 2, 3).

The compartmentalization of Na^+^ into vacuole can alleviate the toxicity of Na^+^ and Cl^−^ in the cytosol and enhance vacuolar osmoregulatory capacity, which could confer salt tolerance of plants (Gaxiola et al., 2001). The vacuolar ATPase (V-ATPase) and pyrophosphatase-dependent H^+^-pyrophosphatase (VP1) as the proton pumps function in establishing and maintaining the electrochemical proton gradient across the tonoplast (Kirsch et al., 1996; Maeshima, 2000). The Na^+^ sequestration process is mediated by the vacuolar Na^+^/H^+^ antiporter (NHX) that is driven by V-ATPase and VP1 (Apse et al., 2003; Bhaskaran and Savithramma, 2011). When *E. salsugineum* plants were treated with NaCl, a salt-induced increase in the EsNHX1 protein expression from PM fractions of roots, and a similar increased expression of the vacuolar H^+^-translocating ATPase E subunits (VHA-E) in leaf tissues were detected (Vera-Estrella et al., 2005; Kant et al., 2006). Taken together, salt stress enhanced H^+^-transport and hydrolytic activity of *E. salsugineum* tonoplast and plasma membrane H^+^-ATPases, and also greatly stimulated the activity of tonoplast Na^+^/H^+^ exchange in *E. salsugineum* leaves and roots (Vera-Estrella et al., 2005; Figures 2, 3).

High-affinity potassium transporters (HKTs) play a key role in reducing Na^+^ toxicity through K^+^ uptake (Rus et al., 2004). The study found that *EsHKT1;2* mRNA is dramatically induced, whereas *AtHKT1* is downregulated upon salt stress (Ali et al., 2013; Figures 2, 3). And *EsHKT1;2*-RNAi lines display a severe potassium deficiency, and are sensitive to high Na^+^ (Ali et al., 2013). *E. salsugineum EsHKT1;2* ectopically expressed in yeast mutants lacking Na^+^ or K^+^ transporters showed a strong K^+^ transporter activity, and selectivity for K^+^ over Na^+^, suggesting EsHKT1;2 act as maintenance of K^+^ uptake under salt stress that supporting the halophytic lifestyle (Ali et al., 2012, 2013; Figures 2, 3). Further, the plants overexpressing *Eutrema EsHKT1;2* in *Arabidopsis athkt1* mutant was proved to be less sensitive to Na^+^ as well as less K^+^ deficiency, compared to the plants overexpressing *AtHKT1* gene in *athkt1* (Ali et al., 2012). Importantly, the K^+^ specificity of EsHKT1;2 protein is based on amino acid differences in the pore of the transporter protein relative to AtHKT1 (Ali et al., 2013). In addition, the transcript level of *EpHKT1;2* from halophytic *E. parvula* (formerly known as *Thellungiella parvula*) increased rapidly in response to high salinity, and the yeast cells expressing *EpHKT1;2* could tolerate high concentrations of NaCl (Ali et al., 2018). Moreover, *Arabidopsis* plants (Col-*gl*) overexpressing *EpHKT1;2* gene accumulated less Na^+^ and more K^+^, and displayed significantly higher tolerance to salt stress when compared to those overexpressing *EpHKT1;1* or *AtHKT1*. Thus, EpHKT1;2 is suggested to mediate tolerance to Na^+^ ion toxicity in *E. parvula*, and is a major contributor to its halophytic nature (Ali et al., 2018; Figure 3).

Besides, the major site of Na^+^ accumulation occurs in old leaves, followed by young leaves and taproots, and the least accumulation occurred in the lateral roots of *E. salsugineum* plants under salt stress (Vera-Estrella et al., 2005). Notably, although most genes in *E. salsugineum* exhibit ~90% identity with *Arabidopsis* genes, the size of *E. salsugineum* transport genes population is ~1.5 times that compared of the corresponding *Arabidopsis* genes (Taji et al., 2008). This suggests that the transporter encoding genes could be remarkably different from their co-orthologs in *Arabidopsis*, and regulate a unique ion transportation system in this halophyte (Taji et al., 2008).

Taken together, these results revealed *E. salsugineum* as a halophyte is able to distribute and store Na^+^ by very strict control of ion movement across both the tonoplast (TP) and plasma membrane (PM) (Vera-Estrella et al., 2005; Figures 2, 3). Correspondingly, *Eutrema* accumulated lower levels of Na^**+**^ and Cl^−^ than *Arabidopsis*, and its rosette leaves displayed more efficient protective mechanisms against Na^+^ metabolic toxicity when plants were exposed to higher salinity stress (M'rah et al., 2007). These works of key Na^+^-transport mechanisms provide some detailed mechanisms analysis underlying salinity tolerance in this halophytic *E. salsugineum*, an *Arabidopsis* relative model system (Vera-Estrella et al., 2005; Figures 2, 3).

## Antioxidants, Redox Balance, and Salt Stress Tolerance

**Figure 4:**
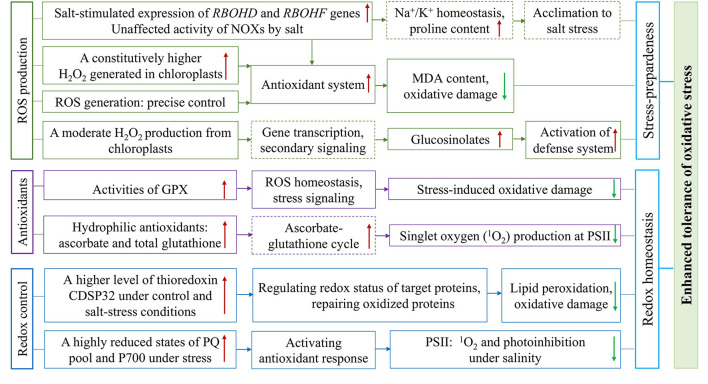
Overview of the constitutively higher expressed antioxidative protection system and effective regulation of redox status in halophytic *Eutrema*. The plasma membrane ROS produced through NADPH oxidases (NOXs), the important components of signal transduction, encoded by *RBOHD* and *RBOHF* genes, is associated with the regulation of Na^+^/K^+^ homeostasis and increased accumulation of proline involved in salt tolerance. The enhanced antioxidant tolerance of *Eutrema* is associated with: (1) A constitutively enhanced H_2_O_2_ generated to keep the antioxidant system up-regulated to preadapted plant to salinity stress, as well as a chloroplastic production of H_2_O_2_ inducing the accumulation of glucosinolates to activate the stress defense system, (2) Antioxidative enzymes such as GPX, and non-enzymatic antioxidants such as ascorbate and glutathione facilitating ROS homeostasis and alleviating damage of singlet oxygen (^1^O_2_) to PSII under salt stress, (3) A higher expression level of thioredoxin CDSP32 regulating redox status of target proteins and highly reduced states of PQ pool and P700 facilitating redox homeostasis and lower photoinhibition under salt stress. NOXs, NADPH oxidases; GPX, glutathione peroxidases; PSII, photosystem II; PQ, plastoquinone.

A common reaction of plants to salt-triggered osmotic stress and ionic imbalance is to increase the levels of reactive oxygen species (ROS; Bose et al., 2014; Kumar et al., 2017). The ROS species of superoxide (${\text{O}}_{2}^{•-}$), hydrogen peroxide (H_2_O_2_), hydroxyl radical (OH^•^), and singlet oxygen (^1^O_2_) are generated constantly as an unavoidable consequence of aerobic metabolism (Møller et al., 2007). The primarily negative effects of ROS molecules on the cells include lipid peroxidation, protein denaturation, and DNA damage (Møller et al., 2007). On the other hand, ROS as universal signaling metabolites has been perceived to regulate plant growth and development, as well as defense against biotic and abiotic stresses (Foyer et al., 2017; Czarnocka and Karpiński, 2018; Fichman and Mittler, 2020). Usually, low levels of H_2_O_2_ could modulate many biological and physiological roles, whereas a high concentration of H_2_O_2_ will cause damage to cellular structures resulting in severe injury to plants (Ozyigit et al., 2016). Thereby, the salt tolerance of halophytes seems to be associated with their ability to control the redox balance (Ozgur et al., 2013; Bose et al., 2014; Surówka et al., 2019; Pilarska et al., 2021; Figures 2, 4).

The H_2_O_2_ checked in *A. thaliana* and *E. salsugineum* plants treated with H_2_O and the salt solution containing 150 and 300 mM NaCl revealed that, in the chloroplasts of salt cress mesophyll cells, electron-dense perhydroxide cerium precipitates were detectable in control, and more pronounced visible dark spots found after salt stress (Pilarska et al., 2016). In contrast, the chloroplasts in *A. thaliana* leaves displayed no perhydroxide deposits in the control condition, while salt stress associated with H_2_O_2_ accumulation was mainly visible at the edges of grana in thylakoids (Pilarska et al., 2016). This result suggested that a constitutively enhanced H_2_O_2_ generated in chloroplasts may be a crucial component of stress-preparedness of this halophyte (Pilarska et al., 2016; Figures 2, 4). Similarly, increased content of H_2_O_2_ in leaves was documented in *E. salsugineum* than in *A. thaliana*. Meantime, the salinity-induced thylakoid swelling was detected only in *Arabidopsis* chloroplasts, while no signs of such destruction occurred in *E. salsugineum* chloroplasts (Pilarska et al., 2016). However, a very low level of MDA was detected, in spite of the higher ROS availability in *E. salsugineum* leaves, indicating a low extent of oxidative damage to membrane lipids (Pilarska et al., 2016). Thus, it is suggested that enhanced leakage of H_2_O_2_ from plastids keeps the antioxidant system up-regulated to make plants preadapted to the salinity stress (Pilarska et al., 2016; Figures 2, 4). The effect of H_2_O_2_ signaling not only depends on its type, but also on the site of its origins such as chloroplasts, mitochondria, and peroxisomes in the cell (Sewelam et al., 2014; Pilarska et al., 2021). The comparison of *A. thaliana* and *E. salsugineum* demonstrated that the latter was capable of enhancing the production of H_2_O_2_ under control and stress conditions (Wiciarz et al., 2015; Pilarska et al., 2016, 2021; Figures 2, 4). These studies indicate a different regulation of ROS formation in the two relative species, and the precise control of ROS generation may contribute to plant stress responses (Pilarska et al., 2021).

In the plasma membrane, ROS are synthesized by NADPH oxidases (NOXs), termed respiratory burst oxidase homologs (RBOHs; Kaur et al., 2018), and NOXs is considered an important component of signal transduction under salt stress (Pilarska et al., 2021). *Arabidopsis NOX* genes, *RBOHD* and *RBOHF*, are the main genes encoding NADPH oxidases associated with acclimation to salinity (Ma et al., 2012; Pilarska et al., 2021). After *E. salsugineum* was exposed to 5 days of salinity, a salt-stimulated expression in *RBOHD* and *RBOHF* genes in leaf tissues was revealed (Figures 2, 4), while their orthologous genes expression in *Arabidopsis* decreased (Pilarska et al., 2021). The ROS produced by RBOHD and RBOHF is associated with the regulation of Na^+^/K^+^ homeostasis under salinity (Chung et al., 2008; Ma et al., 2012), as well as the increased the accumulation of proline involved in salt tolerance (Ben Rejeb et al., 2015; Pilarska et al., 2021; Figure 4). Moreover, the different salt-induced expression patterns of *RBOHD* and *RBOHF* genes between the *E. salsugineum* and *A. thaliana* point to, in leaves of the halophytic *Eutrema*, the two *NOX* genes (*RBOHD* and *RBOHF*) be involved in the late acclimation response to ionic and osmotic stress (Pilarska et al., 2021; Figure 4). Further, the total activity of NOXs in *E. salsugineum* was demonstrated to be unaffected by severe salt stress (Figure 4), whereas a declined activity was seen in *A. thaliana* (Pilarska et al., 2021). And the prolonged mild salinity did not lead to obvious changes in the activity of NOXs in leaves, indicating that the acclimation to salinity in the halophytic *E. salsugineum* was in connection with the maintenance of the basal activity of NOXs (Pilarska et al., 2021; Figure 4). This opinion is in agreement with the study from Srivastava et al. (2015), that the total NOXs activity in the halophyte *Sesuvium portulacastrum* was unaffected, whereas a significantly decreased NOXs activity in the glycophyte *Brassica juncea* (Srivastava et al., 2015).

Antioxidative enzymes play an important role in scavenging toxic radicals in the different organelles of plants such as chloroplasts, cytosol, mitochondria, and peroxisomes, particularly in environmental stress such as salinity (Rajalakshmi and Parida, 2012). In *E. salsugineum*, the enzymatic antioxidant systems could contribute to its extremely high level of tolerance to salt, drought, cold, and oxidative stresses (Gao et al., 2014). Glutathione peroxidases (GPXs) catalyze the reduction of H_2_O_2_ and organic hydroperoxides into the water and correspondingly alcohols, using reduced glutathione (GSH). Plant GPXs play essential roles that are not only involved in ROS homeostasis, stress signaling, and protecting plants from stress-induced oxidative damage to membrane and protein, but also in plant growth and development (Gao et al., 2014; Ozyigit et al., 2016). The gene and protein expression profiles of eight GPXs identified in *E. salsugineum* displayed that *GPX5, GPX7*, and *GPX8* genes were upregulated in both leaves and roots upon salt stress (Figures 2, 4), while *GPX1, GPX2*, and *GPX3* were only upregulated in roots under salt stress; *GPX1, GPX3, GPX4, GPX7* genes in leaves and almost all GPX genes in roots were up-regulated under osmotic stress (Gao et al., 2014). An enhanced GPX activity (Figures 2, 4), but decreased activity of ascorbate peroxidase (APX) were revealed in *Eutrema* plants (Pilarska et al., 2016). These studies documented that, the different members of the *GPX* gene family were modulated in specific environmental stresses and/or in different tissues, indicating the important roles of EsGPXs in salt and osmotic stress response of *Eutrema* plants (Gao et al., 2014).

Thioredoxin is a small oxidoreductase functioning as a hydrogen donor of target proteins and is considered a specific marker for assessment of oxidative stress (Holmgren, 1985; M'rah et al., 2007). Thioredoxin CDSP32 participating in the repair of oxidized proteins during environmental constraints is regarded as a critical component of the defense system against lipid peroxidation and oxidative damage (Rey et al., 2005; Dos Santos and Rey, 2006; M'rah et al., 2007). It was reported that potato (*Solanum tuberosum*) CDSP32 exhibited a substantial accumulation in chloroplast stroma and preserved chloroplastic structures against oxidative injury under drought and oxidative stresses (Broin et al., 2000). In *E. salsugineum*, thioredoxin CDSP32 was found to be higher in abundance than that in *A. thaliana* under control and salt-stress conditions (Figures 2, 4), and strongly diminished in salt-treated *Arabidopsis* plants (M'rah et al., 2007). Furthermore, the magnitude of electrolyte leakage and level of lipid peroxidation were modest in *E. salsugineum*, and very remarkable in *Arabidopsis* (M'rah et al., 2007).

In addition, a significantly increased hydrophilic antioxidants (ascorbate and total glutathione), but no changed lipophilic antioxidants existed in the halophytic *E. salsugineum* when exposed to 300 mM NaCl (Figures 2, 4). In contrast, the glycophyte *Arabidopsis* showed a decreased ascorbate content but increased lipophilic antioxidants (α-tocopherol, plastochromanol, and hydroxy-plastochromanol) due to 150 mM NaCl (Wiciarz et al., 2018). The redox states of plastoquinone (PQ) and P700 were differently regulated by salinity between the halophyte *E. salsugineum* and glycophyte *A. thaliana*, which was documented by effectively avoiding harmful singlet oxygen (^1^O_2_) in the PSII of *Eutrema* (Figures 2, 4), whereas an increased oxidation extent was observed in *Arabidopsis* (Wiciarz et al., 2018). Moreover, *E. salsugineum* has a very efficient antioxidant protection in which glucosinolates may play a specific role (Pilarska et al., 2016; Figure 4).

To sum up, these researches proposed that the salt tolerance of halophytic *Eutrema* species is associated with constitutively expressed protection systems against oxidative stress such as GPX and thioredoxin CDSP32, as well as the ability to sustain a highly reduced state of PQ pool and P700, to alleviate the damage of PSII under salt stress (Figures 2, 4).

## Preformed Metabolites and Osmotic Adaptation

Accumulation of osmoprotectants is one of the defense mechanisms for plants to cope with abiotic stresses (Rajalakshmi and Parida, 2012; Zhao et al., 2020). Several studies have shown that plant metabolism is strongly impacted by salt stress (Flowers and Colmer, 2008; Bartels and Dinakar, 2013; Lee et al., 2016). The metabolites positively correlating with salt stress are reported to participate in the underlying mechanisms including cell-wall remodeling (hydroxyproline), osmoprotectant and storage (proline and sucrose), and photorespiration (glycine and serine; Lugan et al., 2010). The *E. salsugineum* had much higher levels of most metabolites than *A. thaliana* under the salt or osmotic stress treatment (Figure 2), despite the fact that the same metabolic pathways were regulated by salt stress in both species (Lugan et al., 2010). Importantly, significant differences observed in *E. salsugineum* and *A. thaliana* were also associated with the physicochemical properties of their metabolomes, such as water solubility and polarity (Lugan et al., 2010). Comprehensive quantification of organic and mineral solutes documented relative stability of the total solute content in the two species under various treatment conditions (Lugan et al., 2010). But *E. salsugineum* could cope with osmotic stress by tolerating dehydration *via* the metabolic configuration to lend itself to osmoprotectant rather than osmo-adjustment strategy (Lugan et al., 2010; Figure 2). Moreover, the leaves of *E. salsugineum* have a constitutively lower water content than in *A. thaliana*, and have the ability to lose more water without losing turgor, which could contribute to the maintenance of a water potential gradient between the soil and the plant (Lugan et al., 2010; Figure 2). Taken together, salt stress markedly decreased the osmotic potential of shoots, increased total solute levels to sustain a water potential gradient with the environment (Figure 2).

Furthermore, several studies supported the constitutively higher levels of proline, inositols, sugars, and organic acids in *E. salsugineum*, which were interpreted as metabolic stress-anticipation (Gong et al., 2005; Lee et al., 2016; Figure 2). The salt responses of most metabolites in *E. salsugineum* Yukon took place at 200 mM NaCl, and a few additional changes were observed between 200 and 500 mM (Lee et al., 2016).

The compatible osmolyte proline's accumulation is an important marker of salt tolerance in plants (Verbruggen and Hermans, 2008). Salt cress accumulated a markedly higher amount of proline than that of Arabidopsis under normal growth conditions, thus suggesting that the characteristic of extreme tolerance of salt cress to high salinity is due in part to the overaccumulation of proline under unstressed conditions (Taji et al., 2004; Figure 2). Likewise, Kant et al. (2006) demonstrated that *E. salsugineum* contained higher unstressed levels of proline than *Arabidopsis*, while under salt stress, *Eutrema* accumulated more proline mainly in shoots (Kant et al., 2006). The evaluation of salinity stress tolerance of eight different *Eutrema* accessions revealed that Tuva and Buriatia classified as salt susceptible ecotypes treated at 500 mM NaCl had seven-fold higher proline than that in Altai 1 and Altai 2 identified as salt tolerant ecotypes when treated with a similar concentration of NaCl (Gandour et al., 2019). Another study revealed that phenotypic and metabolic adaptive plasticity observed in *E. salsugineum* in different growth conditions could be an inherent trait endowing the flexibility of the halophyte that is required for an extremophile lifestyle (Guevara et al., 2012; Kazachkova et al., 2013).

In summary, these presented data support the markedly higher basal metabolite levels (proline and secondary compounds) in *E. salsugineum* than *Arabidopsis* in normal growth conditions (Figure 2), which is an important characteristic of salt tolerance of salt cress plants. The metabolic pre-adaptation of this halophyte could in part be attributed to the decreased osmotic potential in shoots and increased total solute levels, as well as the physicochemical properties of the metabolites such as water solubility and polarity (Figure 2).

## Gene Expression and Regulation

Salt cress plants can grow in high salinity coastal areas, even in a 500 mM NaCl medium, but they do not have salt glands or other significant morphological alterations either before or after salt adaptation (Inan et al., 2004; Taji et al., 2004). Thus, the salt-tolerant halophyte *E. salsugineum* and salt-sensitive glycophyte *A. thaliana* are considered to use common mechanisms to cope with salinity, and the differences in salt tolerance between the two species can be mainly attributed to the changes in the regulation of a basic set of salt tolerance genes (Kant et al., 2006).

The gene expression profiles in *E. salsugineum* (Shandong ecotype) using an *Arabidopsis* full-length cDNA microarray revealed that, in contrast to *Arabidopsis*, only a few genes were induced by 250 mM NaCl stress, and many known stress-inducible genes such as *Fe-SOD, P5CS, PDF1.2, AtNCED, P-protein, b-glucosidase*, and *SOS1* were expressed at high levels in salt cress even in the absence of stress (Taji et al., 2004; Figure 2). The higher expression of the *P5CS* gene, encoding a key enzyme of proline biosynthesis, is in accordance with a much higher level of accumulated proline in salt cress than did Arabidopsis under normal growing conditions (Taji et al., 2004). Likewise, a comparison of transcript profiles between *Arabidopsis* at 150 mM NaCl and *Eutrema* (Shandong) exposed to 250 mM NaCl, using a microarray platform and *Arabidopsis* homologous gene probes, demonstrated that 40% shared and 60% distinguished regulated genes existed in the halophyte and the glycophyte (Gong et al., 2005). The combined transcript and metabolite analyses notably pointed toward a stress-anticipatory preparedness in *E. salsugineum* (Gong et al., 2005). In comparison with a great activation of transcription detected in *A. thaliana*, only a slight change in gene expression was found in *E. salsugineum* after stress treatment (Taji et al., 2004; Wong et al., 2006). Collectively, the analysis of expression profiling suggested that the stress-inducible signaling pathways are constitutive and active in salt cress even in the absence of stress (Figure 2), which is considered an important strategy for the extreme salt tolerance of this halophyte (Taji et al., 2004; Gong et al., 2005).

Also, comparative proteomics of salt responses between *A. thaliana* and *E. salsugineum* revealed more changes in protein abundance in *Arabidopsis* than in *Eutrema* (Pang et al., 2010). This indicates that the adjustment of metabolism and activation of the already existing enzymatic machinery could serve as a faster and more efficient strategy to cope with salt stress than the synthesis of new proteins (Pilarska et al., 2016; Figure 2). Furthermore, *E. salsugineum* exposed to 300 mM NaCl was used to investigate global transcriptional changes, and genes of several pathways mainly involved in lignin biosynthesis and accumulations of soluble sugars, as well as genes in autophagy and peroxisome pathways, were significantly enriched in the halophyte, suggesting these upregulated genes could contribute to salt tolerance of the halophytic *Eutrema* (Li et al., 2021).

In addition, the cDNA microarray containing 3,628 stress-induced *E. salsugineum* (Yukon ecotype) unique sequences were used to investigate the gene expression-profiling between Yukon and *Arabidopsis* subjected to saline, simulated drought, and cold stress as well as recovery from water deficits. The result revealed that, among 154 differentially regulated transcripts obtained in all studied conditions, only six genes responded to all three stresses of salinity, drought, and cold (Wong et al., 2006). The low overlapping genes indicated an extensive divergence that exists in the responses of *Eutrema* Yukon to different stresses (Wong et al., 2006). There were relatively few transcripts responding to high salinity in this halophytic *E. salsugineum*, in contrast with *A. thaliana* (Wong et al., 2006). Intriguingly, in addition to activating the expression of some well-known stress-responsive genes, a large number of biotic stress-related genes under salinity and drought treatments were found to be down-regulated, suggesting a precise defense strategy to different environmental stresses in *E. salsugineum* (Figure 2), thereby maximizing its survival potential *via* conserving energy and resources (Wong et al., 2006).

MicroRNAs (miRNAs) are known to regulate gene expression by cleavage of complementary target mRNA, repression of translation of target mRNA, and transcriptional silencing of target mRNA (Sunkar and Zhu, 2004; Mittal et al., 2016). Solexa sequencing platform together with microarray and qRT-PCR was used to identify salt-stress responsive miRNAs in *E. salsugineum*. The differential response of the conserved high abundance miR166f, miR168a, miR408, and miR408-5p to salt stress suggests that they may play a key role in salt tolerance of *Eutrema* (Zhang et al., 2013; Figure 2). In agreement with this result, miR166, miR168a, and miR408 were all previously consistently demonstrated to be responsive to stress conditions, including salt and drought stress (Liu et al., 2008; Kong et al., 2010; Trindade et al., 2010; Zhou et al., 2010; Li et al., 2012; Figure 2). Another research on the large-scale characterization of *E. salsugineum* miRNAs and analysis of potential targets demonstrated that 11 conserved miRNA families including miR160, miR161, miR162, miR164, miR171, miR319, miR390, and miR827, and four novel miRNAs displayed a significant response to 300 mM NaCl stress (Wu et al., 2016). Importantly, *E. salsugineum* miR408 was strongly induced by 200 mM NaCl treatment, and *Arabidopsis* miR408 was induced by cold and mannitol, but not by salt (Liu et al., 2008; Zhang et al., 2013). The higher expression of miR408 leads to improved tolerance to salinity, cold and oxidative stress in *Arabidopsis* (Ma et al., 2015). Therefore, it is suggested that miR408 could play a critical role in the salt tolerance of *E. salsugineum* (Figure 2).

Long non-coding RNAs (lncRNAs) are implicated in the regulation of gene expression at the level of chromatin modification, transcription, and post-transcriptional processing, and are suggested to play an important role in a wide range of biological processes, such as genomic imprinting, chromatin remodeling, transcriptional activation, transcriptional interference, and cell cycle (Mercer et al., 2009; Sun et al., 2018). Recently, lncRNAs were proposed to play an important role in response to abiotic and biotic stress including a significant role in plant salt stress response (Gai et al., 2018). The leaf transcriptomes of Yukon and Shandong *E. salsugineum* plants subjected to a progressive, two-stage drought treatment were analyzed, and 1,007 lncRNAs were detected. Among them, 8% were only expressed in stress-treated plants, which suggested a documented association between lncRNA expression and stress (Simopoulos et al., 2020).

In conclusion, these analyses of gene expression profiles based on the microarray platform and transcriptome in *E. salsugineum* provide useful global insight for elucidating the underlying salt tolerance mechanisms that distinguish it from *Arabidopsis* and help our more in-depth analyzing the extreme stress tolerance in this halophyte species. The investigation of the patterns of miRNA accumulation and target gene analysis revealed the possible roles of miRNAs in the adaptive response of *E. salsugineum* to salt stress (Figure 2), which enables us to access the salt tolerance mechanism of this halophyte from the perspective of small RNA regulation. However, research on the role of lncRNAs in salt resistance of plants is in its infancy, and the function and specific mechanism of action of the lncRNAs in salt tolerance of salt cress await to be studied and explored.

## Candidate Genes for Salt Tolerance

Halophytes have the potential to serve as a rich repository for genes to study abiotic stress mechanisms in plants (Rajalakshmi and Parida, 2012). Based on the various plant defense mechanisms adopted by halophytes, many groups have identified some genes functioning in tolerance to salinity *via* expressing them in transgenic systems (Rajalakshmi and Parida, 2012). In *E. salsugineum*, some important candidate genes for salt tolerance have been identified.

### Aquaporins Genes *AQPs*

The exposure of plants to abiotic stresses challenges the plant water status and triggers highly specific hydraulic responses. Aquaporins (AQPs), a large family of channel proteins (Maurel et al., 2009; Wang et al., 2014), could selectively transport water molecules across the cell membrane that function in the plant water balance (Martre et al., 2002; Li et al., 2011, 2014; Qin et al., 2019). Plant *AQP* genes respond to diverse abiotic stresses such as salinity and drought (Alexandersson et al., 2005; Guo et al., 2006), and play important functions in the responses to environmental stimuli (Maurel et al., 2008; Aroca et al., 2012; Wang et al., 2014).

In *Arabidopsis*, the PIPs (plasma membrane intrinsic proteins) aquaporins were well-characterized to be involved in the regulation of root hydraulic conductivity (*L*p_r_) (Javot et al., 2003; Tournaire-Roux et al., 2003). It has been reported that *Arabidopsis* PIP2;1 and PIP2;2 contribute to approximately 40% of the root hydraulic conductivity (Sutka et al., 2011; Péret et al., 2012), and the modification and localization of PIP aquaporins and root hydraulic conductivity could be regulated by the salt stress (Boursiac et al., 2008; Prak et al., 2008; Martinière et al., 2012; Qin et al., 2019). In *E. salsugineum* (*T. halophila*) seedlings, expression of the *EsPIP1* gene in the shoot and root tissues were induced by high salinity. Overexpression of the *EsPIP1* gene in rice markedly enhanced the tolerance of transgenic plants to salt stress *via* regulating the osmotic potential, accumulation of organic small molecules substances, and the ratio of K^+^/Na^+^ in the plant cells (Qiang et al., 2015). Moreover, EsPIP1 protein was shown to specifically interact with EsPIP2 and a non-specific lipid-transfer protein 2, suggesting that *E. salsugineum* EsPIP1 probably plays key roles in the response to multiple external stimuli, and participates in various physiological processes when plants subjected to salt stress (Qiang et al., 2015). Several *E. salsugineum* PIPs were observed to have a more stable expression in the roots compared with that in the shoots under salt stress conditions, and this specific expression pattern of PIP aquaporins is suggested to improve their ability to maintain a higher level of water transport activity in the roots, and decreasing water transpiration in the shoots (Qin et al., 2019).

The tonoplast intrinsic protein gene *EsTIP1;2* from the halophyte *E. salsugineum* was found to have water channel activity when expressed in *Xenopus* oocytes. And this gene was also able to conduct H_2_O_2_ into yeast cells in response to oxidative stress, and the inhibited growth of *EsTIP1;2* transgenic yeast cells were suggested as a result of H_2_O_2_ influx (Wang et al., 2014). In plants, ectopic overexpression of *E. salsugineum EsTIP1;2* in *A. thaliana* plants revealed an enhanced oxidative stress tolerance of plants after methyl viologen (MV) treatment, accompanied by a decrease of H_2_O_2_ level in the leaf cells (Wang et al., 2014). Meanwhile, *EsTIP1;2* could facilitate the entry of Na^+^ ions into plant cell vacuoles under high salinity conditions, through an indirect process (Wang et al., 2014). In all, the tonoplast AQP gene *EsTIP1;2*, was induced by multiple external stimuli, and a significantly elevated tolerance to salt, drought, and oxidative stress was observed in *EsTIP1;2* transgenic *Arabidopsis* plants (Wang et al., 2014). This suggested that *EsTIP1;2* could act as a multifunctional contributor to the survival of *Eutrema* plants in highly stressful habitats *via* mediating the conduction of both H_2_O and H_2_O_2_ across membranes.

To sum up, the *E. salsugineum* aquaporin gene *EsPIP1;4* (*ThPIP1*) or *EsTIP1;2* (*TsTIP1;2*) overexpressed in heterologous species displayed an enhanced plant abiotic stress tolerance in transgenic plants (Wang et al., 2014; Qiang et al., 2015). These data enhance our understanding of the water balance regulation mechanisms involved in plant water status and root hydraulic conductivity, as well as the responses of aquaporin families to salt stress, in this halophyte.

### Late Embryogenesis Abundant Genes (*LEAs*)

Late embryogenesis abundant (LEA) proteins described as multi-functional stress proteins that play active and significant roles in the stress resistance of plants *via* their function in defencing against dehydration caused by drought and salt (Close, 1996; Tunnacliffe and Wise, 2007). When plants are exposed to salinity, drought, or cold stresses, LEA2 protein members typically accumulated in dehydrating plant seeds and/or in tissues (Veeranagamallaiah et al., 2011), while dehydrins (DHNs) referred to as RAB proteins could be induced in vegetative tissues (Hundertmark and Hincha, 2008; Zhang et al., 2020). The expression of HVA1, a LEA3 protein from barley (*Hordeum vulgare* L.) conferred tolerance of transgenic rice plants to salt stress (Xu et al., 1996). Lee et al. (2013) found that, in *E. salsugineum*, 52 LEA protein sequences represented a high sequence similarity with the orthologs in *Arabidopsis* (Lee et al., 2013). Furthermore, 64 *E. salsugineum* LEA proteins were identified to be classified into eight families, based on transcriptome sequencing, as well as the domain structures, numbers, and composition of conserved repeat motifs in LEAs. The remarkably induced expression of *EsLEA1, EsLEA4, EsLEA6*, and *EsDehydrin* genes was revealed in leaves and/or roots, which implies that the expression of LEA proteins could be one of the important strategies for *E. salsugineum* to deal with salt stress (Li et al., 2021).

*Eutrema salsugineum* (*Eutrema halophilum*) EsEm1 (EhEm1) is a hydrophilic protein and contains three different 20-mer conserved motifs of N-domain, M-domain, and C-domain. *EsEm1*gene was revealed to be categorized into group 1 LEA proteins, and its expression was dramatically induced by salt, dehydration, abscisic acid (ABA), and cold stresses in young seedlings (Xiang and Man, 2018; Table 1). Overexpression of the *EhEm1* gene in rice demonstrated an enhanced tolerance to salt and drought stresses in transgenic plants, which was based on the better germination performances, higher survival rates, more accumulation of proline and increased peroxidase activity, as well as the reduced chlorophyll damage and less accumulation of malondialdehyde (Xiang and Man, 2018). And the *EsEm1* gene overexpressed in rice led to an upregulated transcript levels of several stress-responsive genes of *OsCDPK6, OsCDPK9, OsCDPK13*, and *rab16a* under both salt and drought conditions. In addition, EsEm1 protein could effectively prevent the lactate dehydrogenase (LDH) enzyme from inactivation caused by salt and desiccation (Xiang and Man, 2018). Therefore, it is suggested that the *EsEm1* gene was associated with an enhanced salt and drought tolerance in transgenic rice, possibly through its contribution to the stabilization of LDH activity, and the up-regulation of *OsCDPK* genes (Xiang and Man, 2018). In addition, the LEA protein gene *EsLEA1* (*TsLEA1*, EU365627) was characterized as a salt induced gene, and it could improve the survival of salt-sensitive yeast cells, as well as the salt tolerance of transgenic *Arabidopsis* plants when ectopically expressed (Zhang et al., 2012; Table 1). Taken together, it was suggested that the increased expression of LEA protein families acts as protects plants from dehydrating damage, which probably is one of the outstanding mechanisms for adaptation of salt cress to the salt environment.

## Conclusions and Perspectives

Salt cress (*E. salsugineum* or *E. parvula*), a close relative of the model plant *A. thaliana*, occurs and thrives in harsh environments, thus a number of factors contribute to its salt stress tolerance. First, the duplicated gene pairs, particularly the expansion of important resistance genes such as ion transport-related genes in the genome of *Eutrema* may be considered an important adaptive benefit; the generated sub-functionalization and neo-functionalization of duplicated genes could be factors for salt tolerance of salt cress (Hahn, 2009; Kondrashov, 2012; Monihan et al., 2019; Figure 2). Second, the transcriptional and metabolic comparisons of salt tolerant *Eutrema* and susceptible *Arabidopsis* pointed to a stress-anticipatory preparedness with constitutively high expression of the defense genes and relatively few salt-responsive genes detected, accompanied by preformed osmolyte of proline and secondary compounds, and lending metabolite configuration to fit osmoprotectant. And a precise defense strategy facilitates to conservation of energy that makes this halophyte to adapt various extreme environments (Figure 2). Third, *E. salsugineum* emerging as the halophyte, a major advantage appears to be their exceptional control over Na^+^ influx together with export mechanisms, the ability to coordinate its distribution to various tissues, and efficient sequestration of Na^+^ into vacuoles (Figure 2). Fourthly, the combined numerous studies revealed that the key components of underlying mechanisms in salt tolerance of the halophytic *Eutrema*, which is involved in a stronger antioxidative enzymatic system and substantially higher levels of thioredoxin, as well as the superior ability to sustain a highly reduced states of plastoquinone (PQ) pool and P700 under salt stress; the constitutively expressed protection systems against oxidative stress will facilitate to maintain a relatively stable stomatal conductance and CO_2_ assimilation, and enhanced chlorophyll content to keep almost unaffected net photosynthetic rate (Figure 2). Finally, the stress-related genes such as *EsSOS1, EsEm1*, and *EsTIP1;2* that are expressed in transgenic systems led to enhanced tolerance to salinity stress, which indicates they play critical roles in the salt tolerance of *E. salsugineum*.

Tremendous genetic diversity that exists in the halophytic *Eutrema* can be exploited (Koornneef et al., 2004; Gong et al., 2005; Bohnert et al., 2006). And the comparative and functional genomics from this halophyte species have provided important lessons on stress tolerance. Also, overexpression of some abiotic stress-responsive genes from this halophyte into the transgenic model plants and crops has been achieved limited success. However, due to the fact that abiotic stress tolerance cannot be manipulated by a single quantitative trait locus (QTL), and genes could be a part of several pathways at different weights, thus the analysis of a vast of data is needed to explore the complicated regulating network in plants (Rajalakshmi and Parida, 2012). Considering the multigenic nature of abiotic stress tolerance, researchers are working toward regulating networks with more than one kind of gene implicated in stress tolerance, to improve the efficacies of transgenic systems (Rajalakshmi and Parida, 2012).

Going forward, these genetic variations of *E. salsugineum* are providing valuable resources for further deciphering mechanisms underlying the stress tolerance and local adaptations of this halophytic species (Wang et al., 2021). Moreover, *Eutrema* plants undoubtedly have the potential to serve as a rich repository for genes, and the QTL-based approaches in halophytes may be utilized in breeding for abiotic stress tolerance (Rajalakshmi and Parida, 2012). Herein, we provide the recent scientific advance underlying the halophytic *Eutrema* that makes exploration of these fundamental adaptive mechanisms of salt stress more feasible, and to gain opportunities for improving the production of crops in unfavorable salt environments.

## Author Contributions

QZ conceived the manuscript. QZ and YanZ wrote the manuscript with contributions from CL, CD, HZ, YaoZ, and ZM. CL and CD performed the collection of documents and arrangement of data. All authors have read and approved the final manuscript.

## Funding

This work was supported by the Key Technology Research and Development Program of Shandong (2019GSF107089), the Key Program of Nature Science Foundation of Shandong Province (ZR2020KC018), the Granary Science and Technology Demonstration project in the Bohai Sea (2019BHLC001), and the National Major Science and Technology Project of China (2018ZX08009-10B-004).

## Conflict of Interest

The authors declare that the research was conducted in the absence of any commercial or financial relationships that could be construed as a potential conflict of interest.

## Publisher's Note

All claims expressed in this article are solely those of the authors and do not necessarily represent those of their affiliated organizations, or those of the publisher, the editors and the reviewers. Any product that may be evaluated in this article, or claim that may be made by its manufacturer, is not guaranteed or endorsed by the publisher.
